# Knowledge, Attitudes, and Perceived Risks Related to Diabetes Mellitus Among University Students in Uganda: A Cross-Sectional Study

**DOI:** 10.24248/EAHRJ-D-16-00371

**Published:** 2017-07-01

**Authors:** Brenda Kharono, Ruth Nabisere, Nabyonga Kiddu Persis, Jackie Nakakeeto, Abraham Openy, Sabrina Bakeera Kitaka

**Affiliations:** a School of Medicine, College of Health Sciences, Makerere University, Kampala, Uganda; b Mbarara University of Science and Technology, Kampala, Uganda; c School of Health Sciences, Kampala International University, Ishaka, Uganda; d Gulu University, Gulu, Uganda; e Department of Paediatrics and Child Health, Mulago Hospital, Kampala, Uganda

## Abstract

**Background:**

Diabetes mellitus is on the rise in low-income countries, including Uganda, owing to the ‘westernization’ of individual lifestyles. It remains unanswered whether the majority of university students who are rapidly embracing ‘western’ lifestyles have any knowledge of diabetes or perceive themselves to be at risk of acquiring the disease. The aim of the study was to assess the knowledge, attitudes, and perceived risks related to diabetes mellitus among university students in Uganda.

**Methods:**

This descriptive cross-sectional study was conducted in 4 universities in Uganda from August to November 2013. The data collection tool included questions on risk factors, symptoms, personal risks, and practices to prevent diabetes mellitus. We interviewed 378 university students using pretested self-administered semi-structured questionnaires. Only students who consented to participate in the study were included. Data were entered into EpiData version 3.1 and analysed using SPSS version 18.

**Results:**

Almost all (99%) of the students had knowledge about diabetes mellitus. The majority (83.1%) reported that diabetes mellitus is not completely a genetic/hereditary disease. Only a minority of respondents reported that they should worry about diabetes before 45 years of age. Common symptoms of diabetes reported by the respondents included constant hunger, blurred vision, fatigue, and frequent urination.

**Conclusion:**

Our study revealed that the majority of university students in Uganda had good knowledge about the risk factors and symptoms of diabetes mellitus. The majority also perceived themselves to be at risk of diabetes.

## BACKGROUND

Diabetes mellitus is a group of metabolic disorders sharing the common underlying feature of hyper-glycemia.^1^ The estimated worldwide prevalence of diabetes mellitus among adults was 382 million (8.3%) in 2013,^2^ and is predicted to rise to around 438 million (7.7%) by 2030.^3^ There is likely to be an increase in the number of people living with diabetes mellitus worldwide unless preventive action is taken. Sub-Saharan Africa is reported to be one of the regions with the fastest growing rates of diabetes mellitus in the world. Global estimates anticipate the number of people affected by diabetes to increase by 98% from 12 million (3.8%) in 2010 to 24 million (4.7%) in 2030^2,3^; and in Uganda, the prevalence is expected to rise from 2.2% in 2010 to 3.1% by 2030.^3^

The American Diabetes Association defines diabetes as a group of metabolic diseases characterised by hyper-glycemia, which results from defects in insulin secretion, insulin action, or both. It further classifies diabetes mellitus as either type 1 and type 2.^4^ Type 2 diabetes mellitus is the form of diabetes that results from a combination of resistance to insulin action and an inadequate compensatory insulin secretory response.^4^ Although type 2 diabetes is generally considered a disease occurring primarily in adults, it is now being diagnosed more frequently among the youth.^5^ In developing countries, the people in the middle, productive age of their lives are particularly affected by diabetes. In these countries, about 75% of all people with diabetes are under 65 years old and 25% of all adults with diabetes are younger than 44 years. In contrast, more than half of all people with diabetes in developed countries are older than 65 and only 8% of adults with diabetes are younger than 44 years.^5^

The increase in diabetes mellitus in developing countries like Uganda is triggered by many factors, including the unhealthy diets that encompass consumption of high calories, smoking, alcohol use, and sedentary lifestyles, all of which have been subsequent to increasing urbanisation and socioeconomic development.^6,7^ Similar to other African countries, little effort has been invested in the prevention and management of non-communicable diseases (NCDs) in Uganda, despite an increase in the burden of NCDs in the sub-Saharan region.^8^ NCDs are chronic diseases (tend to be of long duration or for life) and are the result of a combination of genetic, physiological, environmental, and behavioural factors.^8^ The burden of infectious diseases like malaria and HIV/AIDS has over shadowed the ‘silent’ increase of NCDs, diabetes inclusive.^6,8,9^ The World Health Organization reported that over a period of 30 years, the burden of NCDs for developing countries was expected to rise by over 60% by 2020, compared with fewer than 10% in developed countries.^10,11^ The ‘silent increase’ in morbidity due to NCDs is attributed to a lack of knowledge and risk perception of the diseases in question.^8^

The cost of treatment for diabetes mellitus is high; the drugs are fairly expensive and the nature of disease demands long-term treatment. The focus is increasing on the prevention, detection, and effective treatment of diabetes. A high awareness of disease in a population is always instrumental in influencing the behaviour of people, as they could easily perceive themselves to be at risk, and thus work towards avoiding catching the disease in question.^9^ In Uganda, diabetes mellitus is mainly managed by doctors. Medical students participate in community-based education services, and are, therefore, expected to possess basic diabetes knowledge to pass on to their patients and the community.

There is no available literature about the knowledge, attitudes, and perceived risk related to diabetes mellitus among university medical students in Uganda. Because little information is known about the extent to which they perceive themselves to be at risk or how knowledgeable they are about the disease, this study aims to assess the knowledge, attitudes, and risk perceptions of university medical students in Uganda regarding diabetes mellitus.

## METHODOLOGY

This descriptive cross-sectional study was conducted from 18 to 30 November 2013 among students in 4 Ugandan universities – Makerere University, Mbarara University of Science and Technology, Kampala International University, and Gulu University Faculty of Medicine.

### Inclusion and Exclusion Criteria

Participants were eligible if they had a valid university student identity card and were medical students, 18 years of age and above, and willing to provide consent to participate in the study during the allocated data collection period. University staff, students with invalid university identity cards, and those unwilling to provide consent for participation were not eligible for inclusion in the study.

### Questionnaire Development and Pilot Test

Following literature review, a student research team from the 4 universities pre-tested self-administered semi-structured questionnaires at Makerere University 2 weeks prior to the official start date of data collection. The pre-test was done to assess question variation, difficulty, and meaning, while at the same time testing respondent interest and attention. Co-investigators, assisted by 10 data collectors had 3-day training on the data collection procedure. All self-administered questionnaires were prepared in English. After Institutional Review Board approval from the different university institutions, a pilot study was done.

### Study Population and Sampling Technique

Although a total of 385 participants was anticipated, only 378 participants consented and were available to participate in the study at the allocated time. The 378 students were selected by simple randomization. Information about the study was read to them by the data collectors before their written informed consent was sought. The self-administered questionnaires were given to the eligible participants in their respective halls of residence. The distribution was done at times that were convenient for the students and did not conflict with academic activities.

The sample size (385) was determined using the Leslie Kish formula. The sample size was calculated as $=\frac{(z^{2})pq}{d^{2}}$, where n was the sample size; z was the value that corresponds to the 95% confidence, which is 1.96 test statistic; p was the proportion of study participants in the population (0.5); q(1-p) was the probability that the outcome did not occur; and d was the acceptable error to be committed (5%), therefore, n = 385. However, only 378 questionnaires were received from the respondents — giving us a response rate of 98.2% — so this number was used in analysis. The independent variables for analysis were year of study, age, and course, and the dependent variables were knowledge, attitudes, and risk perceptions related to diabetes mellitus.

The first part of the questionnaire addressed sociodemo-graphic data, which included age, sex, course, and year of study. The second part addressed overall knowledge of diabetes mellitus, risk factors, and symptoms. The third part covered risk assessment through family history of diabetes mellitus and predisposing lifestyles, such as eating fast foods, smoking, physical inactivity, and alcohol consumption. The fourth part assessed attitudes and risk perceptions towards acquiring diabetes mellitus.

### Data Analysis

The 378 questionnaires were checked for completeness to ensure collection of quality data. Double data entry into EpiData version 3.1 (Odense, Denmark) was done. Data was then exported to SPSS version 18 (Armonk, New York, USA) for analysis. Data was analysed using frequency and percentages, and SPSS was also used to determine the variation of the independent variables.

### Ethics Statement

The study was approved by the research and ethics committee of Makerere University. Only students who provided written consent for participation were included in the study. The informed consent form was approved by the research and ethics committee of Makerere University, which was signed by each student prior to participation.

## RESULTS

### Sociodemographic Characteristics of Respondents

Among the 378 study participants, 229 (60.6%) were male and 149 (39.4%) were female (Table 1). Almost two-fifths (39.2%) of the study participants were from Makerere University, and the remaining three-fifths were divided almost equally between Mbarara University (20.6%), Gulu University (20.6%), and Kampala International University (19.6%).

**TABLE 1.** Sociodemographic Characteristics of Respondents (N=378)

	Responses	
Respondent Characteristics	n	%
**University**		
Gulu	78	39.2
KIU	74	19.6
MAK	148	20.6
MUST	78	20.6
**Year of Study**		
1	75	19.8
2	74	19.6
3	77	20.4
4	74	19.6
5	78	20.6
**Gender**		
Male	229	60.6
Female	149	39.4

Abbreviations: KUI, Kampala International University; MAK, Makerere University; MUST, Mbarara University of Science and Technology.

Students from all the years in the universities had an almost equal participation in the study. The age range of participants was 18–40 years and the mean age was 22 years. About two-thirds (n=248, 65.6%) of the participants reported not having a family member that had been diagnosed with diabetes mellitus, while one-third (n=129, 34.1%) reported positive history of diabetes mellitus.

### Knowledge of Study Participants Regarding Diabetes Mellitus

From Table 2, almost all (99.2%) the students in all the universities across all years of study had heard about diabetes mellitus. Only 3 students (0.8%) reported not having heard about diabetes – 2 from Makerere University (Year 4) and 1 from Gulu University.

**TABLE 2.** Frequency and Percentage of Knowledge of the Study Participants Related to Diabetes Mellitus (N=378)

Variables	Yes n (%)	No n (%)	I do not know n (%)	Not answered n (%)
Have heard about DM	375 (99.2)	3 (0.8)	n/a	n/a
DM is preventable	323 (85.4)	44 (11.6)	11	n/a
DM is genetic	314 (83.1)	45 (11.9)	19 (5.0)	n/a
DM affects only older people	12 (3.2)	357 (94)	9 (2.4)	n/a
DM can be transmitted from one person to another	14 (3.7)	346 (91.5)	15 (4)	3 (0.8)
**Knowledge on Signs and Symptoms**				
Muscle cramps				
Year 1	25 (6.6)	31 (8.2)	n/a	19 (5.0)
Year 2	34 (9.0)	32 (8.5)	n/a	8 (2.1)
Year 3	26 (6.9)	31 (8.2)	n/a	20 (5.3)
Year 4	29 (7.7)	35 (9.3)	n/a	10 (2.6)
Year 5	25 (6.6)	42 (11.1)	n/a	11 (2.9)
Blurred vision				
Year 1	32 (8.5)	30 (7.9)	n/a	13 (3.4)
Year 2	51 (13.5)	17 (4.5)	n/a	6 (1.6)
Year 3	60 (15.9)	6 (1.6)	n/a	11 (2.9)
Year 4	67 (17.7)	6 (1.6)	n/a	1 (0.3)
Year 5	72 (19.0)	4 (1.1)	n/a	2 (0.5)
Constant hunger				
Year 1	48 (12.7)	9 (2.4)	n/a	18 (4.8)
Year 2	59 (15.6)	11 (2.9)	n/a	4 (1.1)
Year 3	63 (16.7)	7 (1.9)	n/a	7 (1.9)
Year 4	66 (17.4)	5 (1.3)	n/a	3 (0.8)
Year 5	76 (20.1)	1 (0.3)	n/a	1 (0.3)
Increased feeling of tiredness				
Year 1	50 (13.2)	11 (2.9)	n/a	14 (3.7)
Year 2	51 (13.5)	12 (3.2)	n/a	11 (2.9)
Year 3	65 (17.2)	4 (1.1)	n/a	8 (2.1)
Year 4	57 (15.1)	9 (2.4)	n/a	8 (2.1)
Year 5	59 (15.6)	15 (4.0)	n/a	4 (1.1)
Frequent urination				
Year 1	53 (14.0)	13 (3.4)	n/a	9 (2.4)
Year 2	64 (16.9)	5 (1.3)	n/a	5 (1.3)
Year 3	65 (17.2)	7 (1.9)	n/a	5 (1.3)
Year 4	71 (18.8)	3 (0.8)	n/a	0 (0)
Year 5	75 (19.8)	3 (0.8)	n/a	0 (0)
Excessive sweating				
Year 1	29 (7.7)	24 (6.3)	n/a	22 (5.8)
Year 2	44 (11.6)	21 (5.6)	n/a	9 (2.4)
Year 3	41 (10.8)	19 (5.0)	n/a	17 (4.5)
Year 4	41 (10.8)	26 (6.9)	n/a	7 (1.9)
Year 5	49 (13.0)	21 (56)	n/a	8 (2.1)
**Knowledge on Risk Factors**				
Eating fast foods	279 (73.8)	97 (25.7)	n/a	2 (0.5)
Smoking tobacco	228 (60.3)	145 (38.4)	n/a	5 (1.3)
Physical inactivity	294 (77.8)	78 (20.6)	n/a	6 (1.6)
Drinking alcohol	315 (83.3)	61 (16.1)	n/a	2 (0.5)
**Knowledge on Prevention**				
Avoiding sweet foods	194 (51.3)	184 (48.7)	n/a	n/a
Diet modification	266 (70.4)	112 (29.6)	n/a	n/a
Regular exercise	175 (46.2)	203 (53.8)	n/a	n/a

Abbreviations: DM, diabetes mellitus; n/a, not applicable.

Of the participants, the majority (83.1%) reported that genetic factors were responsible for diabetes mellitus causation, about a tenth (11.9%) reported that diabetes mellitus is not a genetic disease, and a minority (5%) said they did not know if diabetes mellitus was genetic in origin.

More than half of the students who participated in the study said that consuming fast foods from restaurants (73.8%), smoking (60.3%), physical inactivity (77.8%), and alcohol consumption (83.3%) can predispose you to getting diabetes.

The majority of students (n=325, 86%) demonstrated good knowledge about the common signs and symptoms of diabetes mellitus, this was demonstrated by participants correctly identifying at least 3 of the signs and symptoms of diabetes. Participants knew that diabetes mellitus may present as excessive hunger (82.5%), blurred vision (74.6%), and frequent urination (86.8%). Only 7 participants demonstrated absolute ignorance about the listed signs and symptoms of diabetes mellitus. Of these, 1 student (Year 2) responded ‘no’ to all the listed symptoms, 4 (3 from Year 1 and 1 from Year 3) did not fully answer that question, 1 (Year 1) responded ‘no’ to frequent urination and did not respond to the other symptoms, while 1 (Year 1) responded ‘no’ to constant hunger, blurred vision, and increased blurred vision. It was noted that this question had the biggest percentage of unanswered sections.

Among the 378 participants, the majority (n=323, 85.4%) reported that diabetes mellitus is a preventable disease, under half (n=44, 11.6%) disagreed, and a minority (n=11, 2.9%) did not know whether or not diabetes mellitus is a preventable disease. According to the respondents, the top 3 ways to prevent diabetes was by avoiding sweet foods (51.3%), diet modification (70.4%), and regular exercise (46.2%).

### Attitudes of Participants Towards Diabetes Mellitus

Almost half (48.1%) of the study participants felt that personal efforts would not help control one's risk of getting diabetes. In general, our results showed that minority (8.9%) of the study participants felt that if one was to get diabetes mellitus, there was not much one could do about it, while the majority (90.8%) thought that one could do something to prevent getting the disease. This correlated with majority (89.6%) of positive responses to the statement that ‘people who make a good effort are less likely to get diabetes’ (Table 3).

**TABLE 3.** Participants’ Attitudes and Perceived Risk Related to Diabetes Mellitus (N=378)

Question	Strongly agree n (%)	Agree n (%)	Disagree n (%)	Strongly disagree n (%)	Not answered n (%)
**Attitude Related to Diabetes Mellitus**					
I have little control over risks to my health	28 (7.4)	55 (14.6)	152 (40.2)	140 (37.0)	3 (0.79)
If I am going to get DM, there is not much I can do about it	10 (2.6)	24 (6.3)	150 (39.7)	193 (51.1)	1 (0.26)
My personal efforts will help control my risks of getting DM	9 (2.4)	182 (48.1)	14 (3.7)	168 (44.4)	5 (1.32)
People who make a good effort to control the risks are much less likely to get DM	140 (37.0)	199 (52.6)	22 (5.8)	15 (4.0)	2 (0.53)
**Perceived Risk Related to Diabetes Mellitus**					
Compared to my age mates, I am less likely to get DM	60 (15.9)	121 (32.0)	143 (37.8)	47 (12.4)	7 (1.85)
I should not worry about DM until I am 45 years old	20 (5.3)	36 (9.5)	140 (37.0)	182 (48.1)	0 (0)
DM could be a big threat to my health	188 (49.7)	130 (34.4)	28 (7.4)	32 (8.5)	0 (0)

Abbreviations: DM, diabetes mellitus.

### Perceived Risk Related to Diabetes Mellitus

A majority (85.1%) of the participants disagreed with the statement that they did not have to worry about diabetes until midway into their fifth decade of life (age 45), while only 14.8% thought otherwise. Nearly half (47.9%) of the 378 students reported they were less likely to get diabetes mellitus compared to their age mates. A majority (84.1%) reported that the disease could be a big threat to their health, while the remaining 15.9% said indicated otherwise (Table 3).

## DISCUSSION

Almost all (99.2%) of the participants in our study knew about diabetes mellitus. These findings are different from what was reported among medical students in Saudi Arabia, where knowledge of the prevalence of the disease among medical students was less than 30%.^12^ A study in Libya, however, reported high knowledge levels (76.7%) related to diabetes mellitus among the medical students.^13^ The Libya study, however, sampled final year medical students whereas our study population sampled students across all the years. Students in their final year are more likely to have more knowledge since they are on their final path to medical practice. The high level of knowledge among our study participants may be due to the compulsory community attachment part of the medical student curriculum that mandates students work in communities and are expected to interact with individuals in the community and evaluate the key health problems within those communities. This practical experience our study participants receive is important because in developing countries general doctors play a very important role in chronic disease management. This is also important because the prevalence of diabetes mellitus is increasing, with sub-Saharan Africa reported to be one of the regions with the fastest growing rates of diabetes mellitus in the world.^2,3^ Our findings were comparable across almost all the medical schools in Uganda, which may be attributed to the similarity of the medical training curriculum in the different medical schools.^14^ In our study, there were more male participants compared to females, reflecting current enrolments in medical school. We did not assess differences in the levels of knowledge between the genders since they attend similar lectures; however, this has been noted as one of the limitations of our study.

The majority (86%) of students had good knowledge about the signs and symptoms of diabetes. Participants knew that diabetes mellitus may present as excessive hunger (82.5%), blurred vision (74.6%), and frequent urination (86.8%), respectively. In contrast to a study by Khan et al. among university students in Ajamn, United Arab Emirates (UAE), students reported excessive eating (36.0%), blurred vision (39.0%), and excessive urination (58.0%) as symptoms of diabetes mellitus.^15^ More than 70% of participants reported hunger, blurred vision, fatigue, and frequent urination as symptoms of diabetes mellitus. In contrast, Kulkani et al. reported that medical students were able to identify the most common symptoms of diabetes mellitus, such as increasing thirst (14%), increasing hunger (8%), and weight loss (6%), although the knowledge levels were also low (6%–34%).^16^ The differences in knowledge levels may be due to differences in prevalence of diabetes mellitus in the different regions, differences in school curricula in the different regions, or differences in health agenda. Seven participants in our study demonstrated absolute ignorance about the signs and symptoms of diabetes mellitus: 5 were from Year 1, 1 from Year 2, and 1 from Year 3. This shows that the level of knowledge probably increases as one proceeds through the years in medical school.

The majority (83.1%) of the respondents reported that genetic factors were responsible for diabetes mellitus causation, a figure higher than the 60% found by Poornima et al.^17^ In a study by Shu Hui et al., a majority of patients were not aware of the causes of diabetes mellitus.^18^ This underscores the importance of health workers having proper knowledge of the causes of diabetes mellitus so they can educate patients accordingly. However, the study by Khan et al. also reported that university students were able to identify genetic factors as risk factors for diabetes mellitus.^15^ Because of the increased prevalence of NCDs, it is likely that this high level of knowledge among students is due to health education campaigns about diabetes mellitus in the media.

Among the 378 respondents, the majority (n=323, 85.4%) reported that diabetes mellitus is a preventable disease, comparable to a study among university students in Ajman, UAE, where 74% of the students reported that diabetes can be prevented or delayed.^15^

Whereas diet modification was reported by the majority (70.4%) of students as a method of diabetes mellitus prevention, avoiding sweet foods was not as highly regarded, with only 51.3% of respondents reporting it as a method of preventing the disease. The respondents further reported that consumption of fast food (73.8%) and smoking (60.3%) would predispose to diabetes mellitus, while a reduction in alcohol (83.3%) would help prevent diabetes mellitus.

Physical activity is a factor that has been reported to reduce mortality and also lead to improvement among patients with diabetes mellitus. Therefore, it is surprising that up to 53.8% of our respondents reported that regular exercise is not important in diabetes mellitus control; this is despite the report that revealed that inactivity predisposes to diabetes mellitus. Similarly, Shu Hui et al. reported in their study that less than 45% of diabetic patients perform regular exercise to improve diabetes mellitus control.^18^ No surprising, a study by Kulkarni et al. among college students reported that only 12% of students reported sedentary lifestyle as a risk factor for diabetes mellitus.^16^

About two-thirds (65.6%) of our respondents did not have a family member with diabetes, regardless of the high knowledge levels reported. A family history of diabetes has been reported to influence the students’ level of knowledge and perception of diabetes.^19^ This is probably because they will be directly involved in patient care, which also involves family health education.

A majority (85.1%) of the respondents disagreed with the statement that they did not have to worry about diabetes until midway the fifth decade of life (age 45) and majority felt there was much one could do to prevent oneself from getting diabetes mellitus. A large percentage (84.1%) reported that diabetes mellitus could be a big threat to their health. Half (50.2%) of the students perceived themselves at more risk of getting diabetes mellitus than their age mates, however, almost the same percentage (50.5%) felt that personal efforts would help control one's risk of getting diabetes. One of the biggest weaknesses of our study was that we did not assess the students’ own practices towards control of diabetes mellitus; this would have illustrated if those who perceive themselves at risk and believe that their personal efforts can help prevent them from contracting diabetes mellitus are actually doing something about it.

Another limitation noted was not ascertaining which students were also patients living with diabetes and comparing this to their level of knowledge of the disease.

## CONCLUSION

Majority of respondents exhibited high levels of knowledge of the causes and symptoms of diabetes mellitus. The respondents also exhibited a positive attitude towards prevention of the disease. A majority further perceived themselves at risk of getting diabetes. However, this study revealed some gaps in the knowledge of medical students concerning the prevention of diabetes mellitus that can be appropriately used in designing the curriculum for the different medical schools. The study also lacks data to address the practices of the students as regards to their perceived risk. To that end, we recommend future studies fully evaluate the practices of university students related to diabetes mellitus.
